# Profile of Histone H3 Lysine 4 Trimethylation and the Effect of Lipopolysaccharide/Immune Complex-Activated Macrophages on Endotoxemia

**DOI:** 10.3389/fimmu.2019.02956

**Published:** 2020-01-10

**Authors:** Vichaya Ruenjaiman, Patcharavadee Butta, Yu-Wei Leu, Monnat Pongpanich, Asada Leelahavanichkul, Patipark Kueanjinda, Tanapat Palaga

**Affiliations:** ^1^Interdisciplinary Graduate Program in Medical Microbiology, Graduate School, and Center of Excellence in Immunology and Immune-Mediated Diseases, Chulalongkorn University, Bangkok, Thailand; ^2^Department of Microbiology, Faculty of Science, Chulalongkorn University, Bangkok, Thailand; ^3^Department of Life Science, National Chung Cheng University, Chiayi, Taiwan; ^4^Department of Mathematics and Computer Science, Faculty of Science, Chulalongkorn University, Bangkok, Thailand; ^5^Institute for Biomedical Sciences, Interdisciplinary Cluster for Cutting Edge Research, Shinshu University, Nagano, Japan

**Keywords:** macrophage, LPS, immune complex, epigenetics, H3K4me3, endotoxemia

## Abstract

Macrophage plasticity is a process that allows macrophages to switch between two opposing phenotypes based on differential stimuli. Interferon γ (IFNγ)-primed macrophages stimulated with lipopolysaccharide (LPS) [M(IFNγ+LPS)] produce high levels of pro-inflammatory cytokines such as IL-12, TNFα, and IL-6 and low levels of the anti-inflammatory cytokine IL-10, while those stimulated with LPS in the presence of the immune complex (IC) [M(IFNγ+LPS+IC)] produce high levels of IL-10 and low levels of IL-12. In this study, we investigated the plasticity between M(IFNγ+LPS) and M(IFNγ+LPS+IC) *in vitro* and compared one of the active histone marks [histone H3 lysine 4 trimethylation (H3K4me3)] between M(IFNγ+LPS) and M(IFNγ+LPS+IC) using murine bone marrow-derived macrophages. We found that in an *in vitro* system, macrophages exhibited functional plasticity from M(LPS) to M(LPS+IC) upon repolarization after 2 days of washout period while IFNγ priming before LPS stimulation prevented this repolarization. Phosphorylation of p38, SAPK/JNK, and NF-κB p65 in M(LPS+IC) repolarized from M(LPS) was similar to that in M(LPS+IC) polarized from resting macrophages. To obtain the epigenetic profiles of M(IFNγ+LPS) and M(IFNγ+LPS+IC), the global enrichment of H3K4me3 was evaluated. M(IFNγ+LPS) and M(IFNγ+LPS+IC) displayed marked differences in genome-wide enrichment of H3K4me3. M(IFNγ+LPS+IC) showed increased global enrichment of H3K4me3, whereas M(IFNγ+LPS) showed decreased enrichment when compared to unstimulated macrophages. Furthermore, M(IFNγ+LPS+IC) exhibited high levels of H3K4me3 enrichment in all *cis*-regulatory elements. At the individual gene level, the results showed increased H3K4me3 enrichment in the promoters of known genes associated with M(IFNγ+LPS+IC), including *Il10, Cxcl1, Csf3*, and *Il33*, when compared with those of M(IFNγ+LPS). Finally, we investigated the impact of M(IFNγ+LPS+IC) on the systemic immune response by adoptive transfer of M(IFNγ+LPS+IC) in an LPS-induced endotoxemia model. The cytokine profile revealed that mice with adoptively transferred M(IFNγ+LPS+IC) had acutely reduced serum levels of the inflammatory cytokines IL-1β and IL-p12p70. This study highlights the importance of epigenetics in regulating macrophage activation and the functions of M(IFNγ+LPS+IC) that may influence macrophage plasticity and the potential therapeutic use of macrophage transfer *in vivo*.

## Introduction

Macrophages are cells of the innate immune system that are found in most tissues. They respond to infection as the first line of defense against pathogens (1). Macrophages play an important role in the immune response and homeostasis with functional diversity, such as inflammation, phagocytosis, metabolism, tissue remodeling, and immunoregulation. However, a key feature of macrophages is functional plasticity (2).

Macrophage plasticity is a process that allows macrophages to switch between two different phenotypes. The functional plasticity of macrophages contributes to the pathogenesis of various diseases, such as cancer, metabolic diseases, autoimmune diseases, and systemic infections (3). In this study, the common framework for macrophage activation nomenclature as proposed by Murray et al. is followed (4). Interferon γ (IFNγ) and lipopolysaccharide (LPS)-stimulated macrophages or classically activated macrophages [M(IFNγ+LPS)] exhibit pro-inflammatory activity, which is characterized by the production of high levels of pro-inflammatory cytokines (5). LPS and immune complex (IC)-stimulated macrophages [M(IFNγ+LPS+IC)] produce large amounts of the anti-inflammatory cytokine IL-10 and low levels of the pro-inflammatory cytokine IL-12 but produce high levels of tumor necrosis factor (TNFα), IL-6 and IL-1β (6, 7). At least *in vitro*, optimal activation of M(LPS+IC) is reported in the presence of IFNγ (8). IL-10 is a key multifunction regulatory cytokine that regulates the immune response during infection and dampens immune hyperactivation (9, 10).

M(IFNγ+LPS+IC) are categorized as non-classically activated macrophages that show immunomodulatory activity due to the increased production of the anti-inflammatory cytokine IL-10 and reduced pro-inflammatory cytokine IL-12 production (6). Transcriptionally, M(IFNγ+LPS+IC) exhibit a distinctive gene expression profile that is unique and does not overlap with that of M(IFNγ+LPS) or M(IL-4) (11). Therefore, M(IFNγ+LPS+IC) have potential in dampening the overt immune response and can be used in cellular therapy. In one study, adoptive transfer of M(IFNγ+LPS+IC) reduced the severity in an animal model of multiple sclerosis (8). In another study, it was reported that mice with adoptively transferred M(IC) showed reducing disease severity in sepsis (11). To induce LPS-stimulated macrophages to produce higher amounts of IL-10, the signaling downstream of IC/FcR can be replaced with other stimuli, such as PGE2 (11). However, the stability of the M(IFNγ+LPS+IC) phenotype upon transfer *in vivo* remains unknown.

In macrophages, the expression of *Il10* is regulated by several transcription factors, including Sp1, ERK and NF-κB (10, 12). We also reported that Notch signaling plays important roles in regulating IL-10 production in M(IFNγ+LPS+IC) (13). FcγR signaling activates Erk and p38 MAPK signaling in M(IFNγ+LPS+IC), resulting in the binding of Sp1 to the *Il10* promoter (14).

Regulation of cytokine production in macrophages is regulated at several levels, such as transcription factor activation, epigenetic regulation and post-transcriptional regulation (2). Epigenetic regulation plays a critical role in influencing long-term plasticity (15). Epigenetics regulate chromatin accessibility at the promoter and regulatory regions by several processes including histone modifications (16).

Histone methylation can be conducive or repressive to gene expression, depending on the locations of the modified amino acids and the type of methylation on the histone tails (17). Activation of Jmjd3, a demethylase that mediate trimethylation on lysine 27 of histone H3 (H3K27), results in increased chromatin accessibility leading to M(IL-4) signature gene expression and is crucial for regulating M(IL-4) polarization (18). Trimethylation on lysine 4 of histone H3 (H3K4me3) on genes encoding cell surface markers and chemokines correlates with the transcriptional activity in monocyte-derived macrophages (19). Together, these results strongly indicate that both H3K4me3 and H3K27me3 play an essential role in polarization and activation in macrophages (18, 19).

The regulation of IL-10 production in M(IFNγ+LPS+IC) by histone modification has been reported, where ERK activation leads to phosphorylation of serine 10 on histone H3 at the *Il10* promoter. This event increases the recruitment of the transcription factor SP-1 to the *Il10* promoter and increases *Il10* expression (20). However, the global profile of an active histone mark H3K4me3 in M(IFNγ+LPS+IC), in comparison to M(IFNγ+LPS), has not been characterized. In addition, whether M(IFNγ+LPS) can be repolarized to phenotypically become M(IFNγ+LPS+IC) has not been examined. This study, therefore, investigated the plasticity of M(LPS) and M(IC) *in vitro*. We next compared the profiles of H3K4me3 between M(IFNγ+LPS) and M(IFNγ+LPS+IC) to gain a comprehensive view of H3K4me3 involvement in the two distinctive macrophage phenotypes. Finally, the effect of M(IFNγ+LPS+IC) on systemic cytokine production was tested in an LPS-induced endotoxemia model by adoptive transfer approach. Gaining insight into the epigenetic involvement in M(IFNγ+LPS+IC) may shed light on the plasticity and stability of the M(IFNγ+LPS+IC) phenotype *in vivo*.

## Materials and Methods

### Mice

Six to eight week-old female C57BL/6 mice were purchased from Nomura Siam International (Bangkok, Thailand). All procedures were reviewed and approved by the Chulalongkorn University animal care and use protocol committee (Approval No. 024/2558). All experiments involving laboratory animals were performed according to the regulations and recommendations of the Institute Animal Care and Use committee at Chulalongkorn University.

### Murine Macrophages

Bone marrow-derived macrophages (BMDMs) were prepared as described elsewhere. Briefly, bone marrow cells were isolated from tibias and femurs by flushing with Dulbecco's Modified Eagle's Medium (DMEM) (HyClone, Logan, UT, USA) supplemented with 10% (v/v) fetal bovine serum (GIBCO, Grand Island, NY, USA), 1% (w/v) sodium pyruvate, 1% (w/v) HEPES, 100 U/ml penicillin, 0.25 mg/ml streptomycin, 20% L929 cell conditioned media and 5% horse serum (all reagents were purchased from HyClone). Cells were cultured for 1 week, and the medium was changed every 3 days. BMDMs were confirmed by detecting the cell surface markers CD11b and F4/80 by flow cytometry and plated in a tissue culture plate 24 h before use.

### Generating M(IFNγ+LPS) and M(IFNγ+LPS+IC)

BMDMs were primed with recombinant mouse IFN-γ (10 ng/ml; BioLegend, San Diego, CA, USA) for 18 h before stimulation unless otherwise specified. M(IFNγ+LPS) and M(IFNγ+LPS+IC) were generated by adding *Salmonella* LPS (100 ng/ml; Sigma-Aldrich, St. Louis, MO, USA) or LPS in combination with IC. IC was prepared by adding a 10-fold molar excess of rabbit anti-OVA IgG to OVA (both from Sigma-Aldrich), and the complex was incubated for 30 min at room temperature (21). IC was used at a 1:100 volume ratio of IC to media for stimulation.

### Repolarization of M(IFNγ+LPS) or M(LPS) to M(LPS+IC)

BMDMs were first polarized to M(IFNγ+LPS) or M(LPS) for 24 h. Cells were washed with warm media and rested in culture media for 2 h or 48 h before repolarization by adding LPS together with IC for M(LPS+IC). Culture media were harvested at 24 h after the secondary stimulation to measure IL-10 and IL-12p70 by ELISA. Resting BMDMs polarized to M(IFNγ+LPS) or M(LPS), M(IFNγ+LPS+IC) or M(LPS+IC) were used as controls, respectively. For Western blot analysis, BMDMs were polarized to M(LPS) for 24 h followed by a washout period of 2 or 48 h. The protein lysates were collected at 0, 5, 15, and 30 min after the secondary stimulation with LPS/IC. As a control, BMDMs were polarized to M(LPS+IC) and the lysates were collected at 0, 5, 15 min.

### Enzyme-Linked Immunosorbent Assay (ELISA)

Culture supernatants from BMDMs treated as indicated were harvested to measure IL-10 and IL-12p70 by using an IL-10 ELISA (BioLegend) and an IL-12p70 ELISA (BD Biosciences, San Jose, CA, USA). ELISAs were performed following the manufacturer's protocol.

### Western Blot Analysis

BMDMs were treated as indicated, and cell lysates were subjected to Western blot. The antibodies used in this study were rabbit anti-phospho-NF-κB p65 (1:2000), rabbit anti-NF-κB p65 (1:4000), rabbit anti-phospho-Akt (1:2000), rabbit anti-Akt (Ser473) (1:4000), rabbit anti-phospho-p38 (1:2000), rabbit anti-p38 (1:4000), rabbit anti-phospho-ERK1/2 (p42/44) (1:2000), rabbit anti-ERK1/2 (p42/44) (1:4000), rabbit anti-phospho-SAPK/JNK (1:2000), rabbit anti-SAPK/JNK (1:4000), and mouse anti-β-actin (1:10000), HRP-conjugated donkey anti-rabbit IgG and HRP-conjugated sheep anti-mouse IgG (1:4000) (all from Cell Signaling Technology, Danvers, MA, USA). The signals were detected by chemiluminescence. In order to quantify the band densities among different samples, Western blot images were subjected to analysis by ImageJ software.

### ATP Luminescence Assay for Cell Viability

BMDMs were primed with IFN-γ followed by LPS stimulation for 24 h. Cell viability was measured using ATPlite 1-step Luminescence Assay (PerkinElmer, Waltham, MA, USA) according to the manufacturer's protocol. The signals were detected by Varioskan LUX Multimode Reader (ThermoFisher Scientific).

### Endotoxemia and Adoptive Transfer of Macrophages

For the *in vivo* adoptive transfer study, 5 × 10^6^ BMDMs were stimulated as described above for 4 h to generate M(IFNγ+LPS+IC) before transfer. Eight week-old female C57BL/6 mice (*n* = 4 per group) were adoptively transferred with 1 × 10^6^ cells of M(IFNγ+LPS+IC) or control unstimulated macrophages per mouse by *i.p*. administration. Three hours later, mice were injected with *E. coli* LPS (Sigma-Aldrich) at a sublethal dose (1 mg/kg body weight, *i.p*. route) to induce endotoxemia. Blood was collected at 1 and 6 h after the LPS challenge and subjected to cytokine measurement by Bio-Plex assay.

### Bio-Plex Assays

Blood serum of the endotoxemia model mice prepared as described above was subjected to multiple cytokine measurement (IL-1β, IL-12p70, IL-6, TNFα, IL-17, IL-10, and IL-4) by using Bio-Plex Pro™ Mouse Cytokine 7-plex Assay (Bio-Rad, Hercules, CA, USA). Assays were performed according to the manufacturer's instructions, and the data were analyzed using Bio-Plex Manager™ software (Bio-Rad).

### ChIP-seq and Data Analysis

BMDMs were polarized to M(IFNγ+LPS) and M(IFNγ+LPS+IC) for 4 h as described above. Cells were fixed and subjected to the SimpleChIP^®^ Enzymatic Chromatin IP Kit (Magnetic Beads) (Cell Signaling Technology) according to the manufacturer's protocol. The antibody used in the ChIP assay was rabbit anti-H3K4me3 or the isotype control (Cell Signaling Technology). ChIP DNA fragments were analyzed using 50 bp single-end sequencing by BGI (Beijing, China). The trimmed sequences were examined for quality by FastQC and aligned to the reference genome by Bowtie2 (more than 97% mapped) (22). Regions of enrichment were identified using MACS 1.4 and MACS2 (23). Circos and Venn diagrams were employed to visualize the designated ChIP enrichment globally. Epigenomic correlation was evaluated by TCOR in EpiMINE (24). CEAS was used to reveal enrichment in *cis*-regulatory regions. IGV and QIRI in EpiMINE were used to visualize and quantify the enrichment in target genes. The possible regulatory signaling pathways were analyzed by enriched KEGG pathway analysis using clusterProfiler (25) and DOSE (26) R packages. Statistical significance was reported as BH-adjusted *p*-value (27, 28).

## Results

### IFNγ Priming and Resting Durations Determined the Plasticity of M(IFNγ+LPS)/M(LPS) for Repolarization to M(LPS+IC)

**Figure 1:**
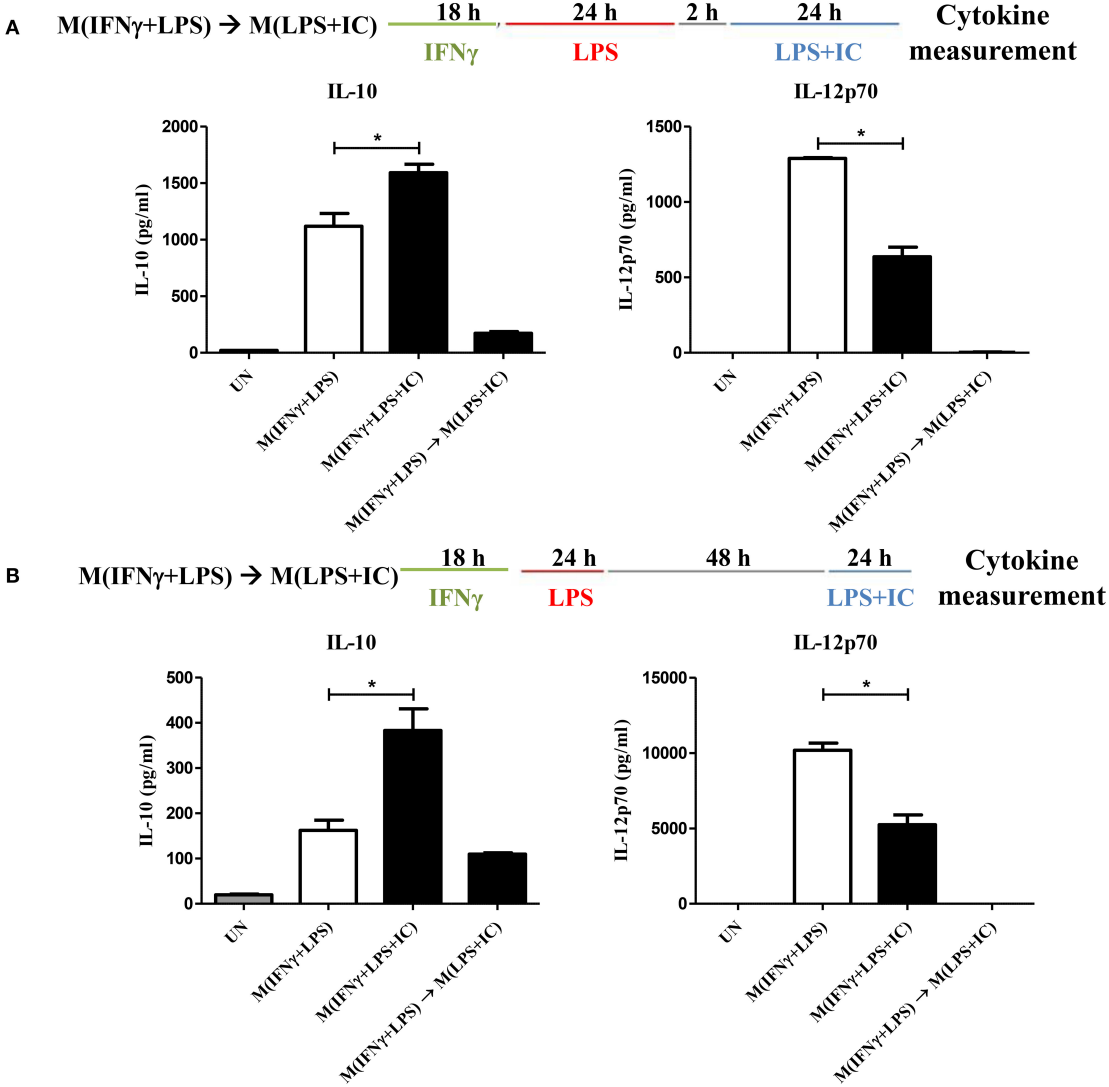
Repolarization of M(IFNγ+LPS) to M(LPS+IC). **(A,B)** The protocol used for stimulation of IFNγ-primed macrophages with LPS and LPS with IC is shown with the washout period of 2 or 48 h. IL-10 and IL-12p70 in the culture supernatant harvested from cells treated as described were analyzed by ELISA. The results indicate the means ± SD of triplicates determined from three independent experiments. ^*^A significant difference at *p* < 0.05.

The plasticity between M1 and M2 macrophages has been reported (5). However, little is known about whether reverse polarization from M(IFNγ+LPS) to M(LPS+IC) is possible. Therefore, we first tested the repolarization plasticity of M(IFNγ+LPS) to become M(LPS+IC), as depicted in Figure 1. The washout periods of 2 or 48 h after the first LPS stimulation were examined. As shown in Figures 1A,B, upon LPS and IC stimulation, M(IFNγ+LPS) failed to increase the level of IL-10 and decrease the level of IL-12p70 as it was expected for M(LPS+IC), whether the washout period between the two stimulation was 2 or 48 h.

**Figure 2:**
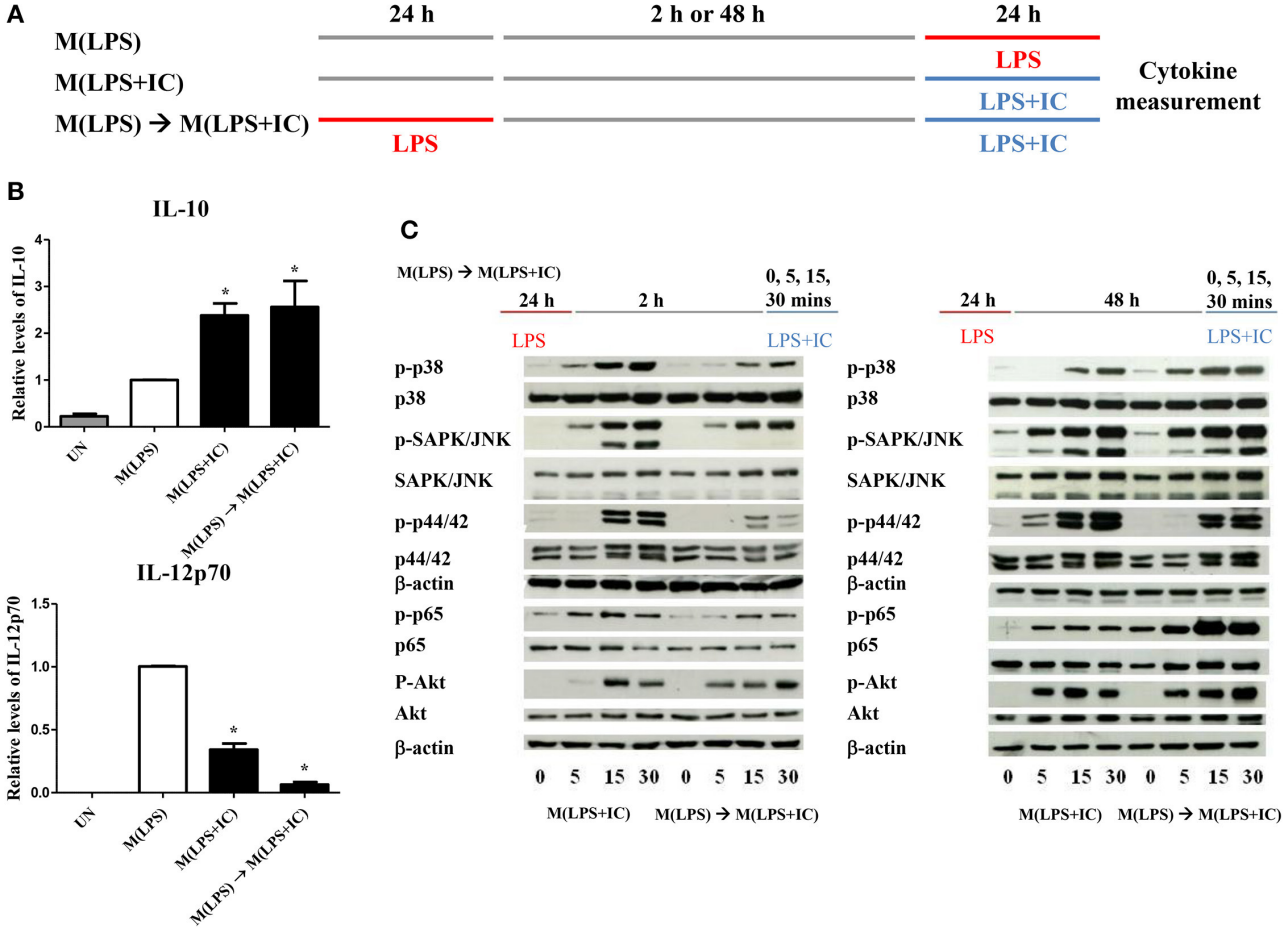
Repolarization of M(LPS) to M(LPS+IC) and signaling downstream of TLR4. **(A)** The protocol used for macrophage stimulation with LPS and LPS with IC is shown. **(B)** IL-10 and IL-12p70 in the culture supernatant harvested from cells treated as described in **(A)** were analyzed by ELISA. The results indicate the means ± SD of triplicates determined from three independent experiments. ^*^A significant difference at *p* < 0.05. **(C)** BMDMs were polarized to M(LPS) and allowed to rest for 2 or 48 h. After re-stimulation with LPS and IC, phosphorylation of MAPKs, NF-κB p65 and Akt was detected in cell lysates by Western blot. β-actin was used as a control. Representative data from one of three independent experiments are shown.

Next, to test whether IFNγ priming prevents the reverse polarization into M(LPS+IC), BMDMs were stimulated with LPS without IFNγ priming and subjected to reverse polarization to M(LPS+IC) with a washout period of 2 or 48 h as depicted in Figure 2A. The results showed that M(LPS+IC) polarized from resting macrophages and M(LPS) with a washout period of 48 h had increased IL-10 and decreased IL-12p70 to a comparable extent (Figure 2B), whereas the shorter washout durations (2, 6, and 24 h) between the primary LPS stimulation and secondary LPS/IC stimulation failed to yield M(LPS+IC) cytokine profiles (Supplementary Figure 1A). A slight decreased in the cell viability between unstimulated cells and M(IFNγ+LPS) was observed (Supplementary Figure 1B). Taken together, this result suggests that IFNγ priming during M(IFNγ+LPS) and a resting duration between the two rounds of stimulation are the key determinants of the plasticity of M(LPS) to be repolarized to M(LPS+IC) *in vitro*.

### Successful Reverse Polarization of M(LPS) to M(LPS+IC) Correlated With the Activation of Early Signaling Pathways in Response to LPS and IC

From the previous results, we found that sufficient resting period between LPS and LPS with IC was important for the observed plasticity. Therefore, we next investigated the early signaling pathway downstream of TLR4. As depicted in Figure 2C, in the condition of 2 h resting between stimulations with LPS and LPS with IC, markedly decreased phosphorylation of MAPK p38, SAPK/JNK and NF-κB p65 at 5, 15, and 30 min was observed in response to LPS with IC when compared to M(LPS+IC) polarized from naïve BMDMs (Supplementary Figure 2). The phosphorylation of Akt and p44/42 was comparable in this condition. In contrast, in the condition where the M(LPS+IC) phenotype was obtained with a resting time of 48 h between the two rounds of stimulation, the phosphorylation of MAPK p38, SAPK/JNK, and NF-κB p65 was comparable with those of M(LPS+IC) from naïve BMDMs (Supplementary Figure 2). These results implied that the defects in early signaling pathways downstream of TLR4 are associated with the inability of M(LPS) to be repolarized to M(LPS+IC), and the increased duration between the two rounds of stimulation rescues this defect, which allows M(LPS) to respond to LPS and IC.

### Increased Global H3K4me3 Enrichment in M(IFNγ+LPS+IC)

**Figure 3:**
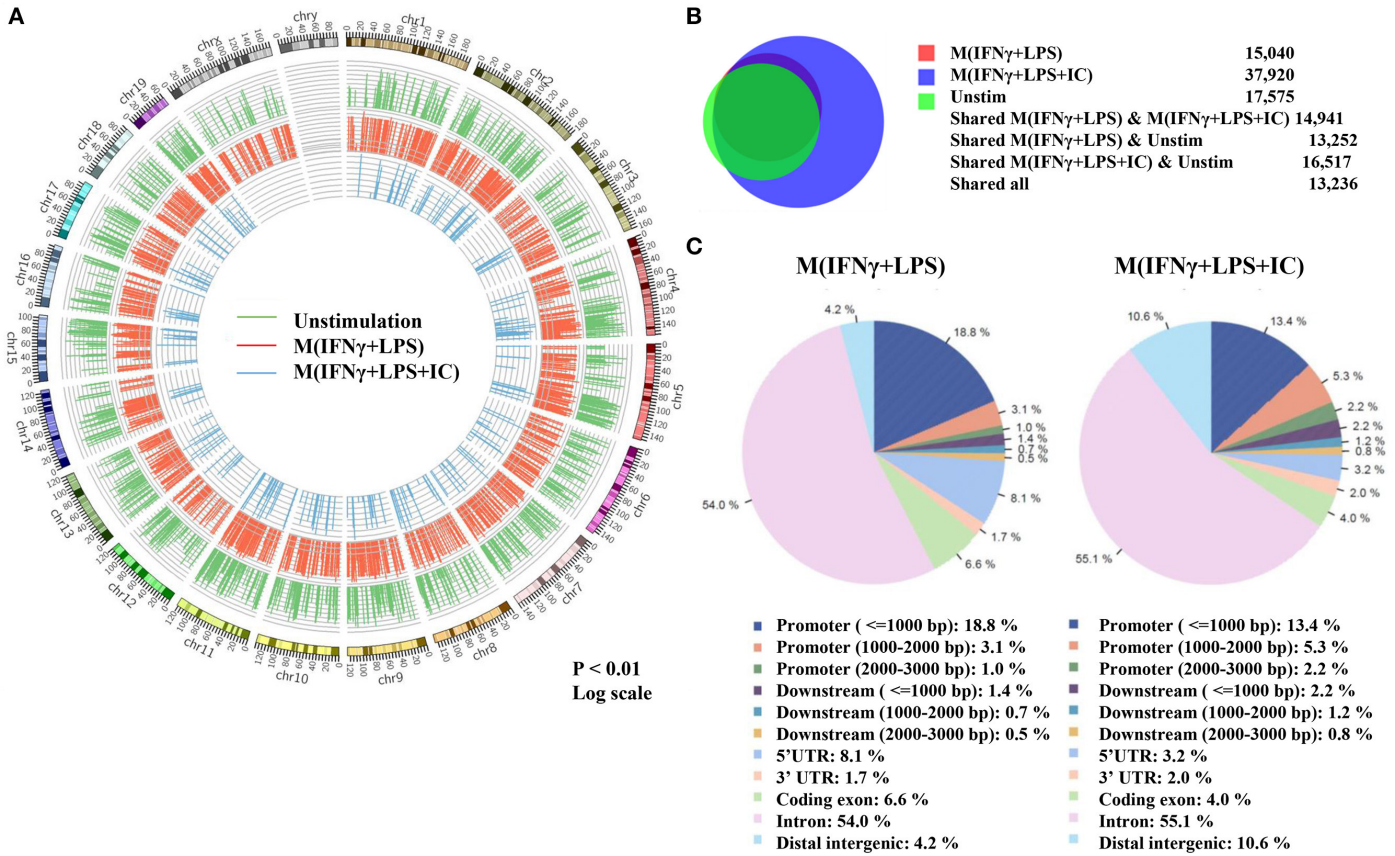
Global enrichment of H3K4me3 in unstimulated macrophages, M(IFNγ+LPS) and M(IFNγ+LPS+IC). BMDMs were primed with IFNγ before stimulation with LPS with or without IC for 4 h. Cells were harvested for ChIP using an anti-H3K4me3 antibody. The ChIP samples were subjected to DNA sequencing. **(A)** Circos plot showing genome-wide H3K4me3 enrichment in unstimulated macrophages, M(IFNγ+LPS) and M(IFNγ+LPS+IC). The positions of log-transformed H3K4me3 enrichment in unstimulated macrophages (green circle), M(IFNγ+LPS) (red circle) and M(IFNγ+LPS+IC) (blue circle) were aligned according to chromosome position in the outer ring. **(B)** The total peaks after identification of H3K4me3 enrichment with MACS 1.4 were used to compare the overlap of H3K4me3 enrichment peaks, and the results are presented in a Venn diagram. **(C)** The *cis*-regulatory element annotation system (CEAS) showed the distribution pattern of H3K4me3 enrichment between M(IFNγ+LPS) and M(IFNγ+LPS+IC). All ChIP-seq results were analyzed from combined RAW files of two independent experiments.

Because the priming with IFNγ and the resting durations between the two stimulation rounds are key determinants of the observed plasticity in the repolarization to M(LPS+IC), we wondered whether epigenetic changes occur during LPS or LPS+IC stimulation after the IFNγ priming. Therefore, epigenetic modifications by one of the active histone marks, H3K4me3, were investigated by ChIP-seq and the profiles were compared among unstimulated macrophages, M(IFNγ+LPS) and M(IFNγ+LPS+IC). A model-based analysis was used to identify significantly enriched H3K4me3 peaks with a *p* < 0.01. Circos plots were used to display global H3K4me3 enrichment among the three groups, and the results revealed clear differences among the samples (Figure 3A). As shown in Figure 3B, we found that the enrichment of H3K4me3 was higher in M(IFNγ+LPS+IC) than in unstimulated and M(IFNγ+LPS), and most H3K4me3 peaks that were enriched in M(IFNγ+LPS) overlapped with those in M(IFNγ+LPS+IC). Next, CEAS was used to identify the distribution of ChIP regions in M(IFNγ+LPS) and M(IFNγ+LPS+IC). M(IFNγ+LPS+IC) showed more distributed peaks in the distal intergenic regions of the promoter (1,000–2,000 bp), while M(IFNγ+LPS) had more peaks distributed in the promoter regions (<1,000 bp) and the coding exons. In both cases, the highest distributed peaks were enriched in the gene bodies, especially the promoter regions upstream <1,000 bp of the transcription start sites (TSSs) (Figure 3C). Taken together, these results reveal that M(IFNγ+LPS+IC) and M(IFNγ+LPS) display similar patterns of global H3K4me3 enrichment profiles, but M(IFNγ+LPS+IC) show increased H3K4me3 enrichment.

### The Correlation of H3K4me3 Peaks Between M(IFNγ+LPS) and M(IFNγ+LPS+IC)

**Figure 4:**
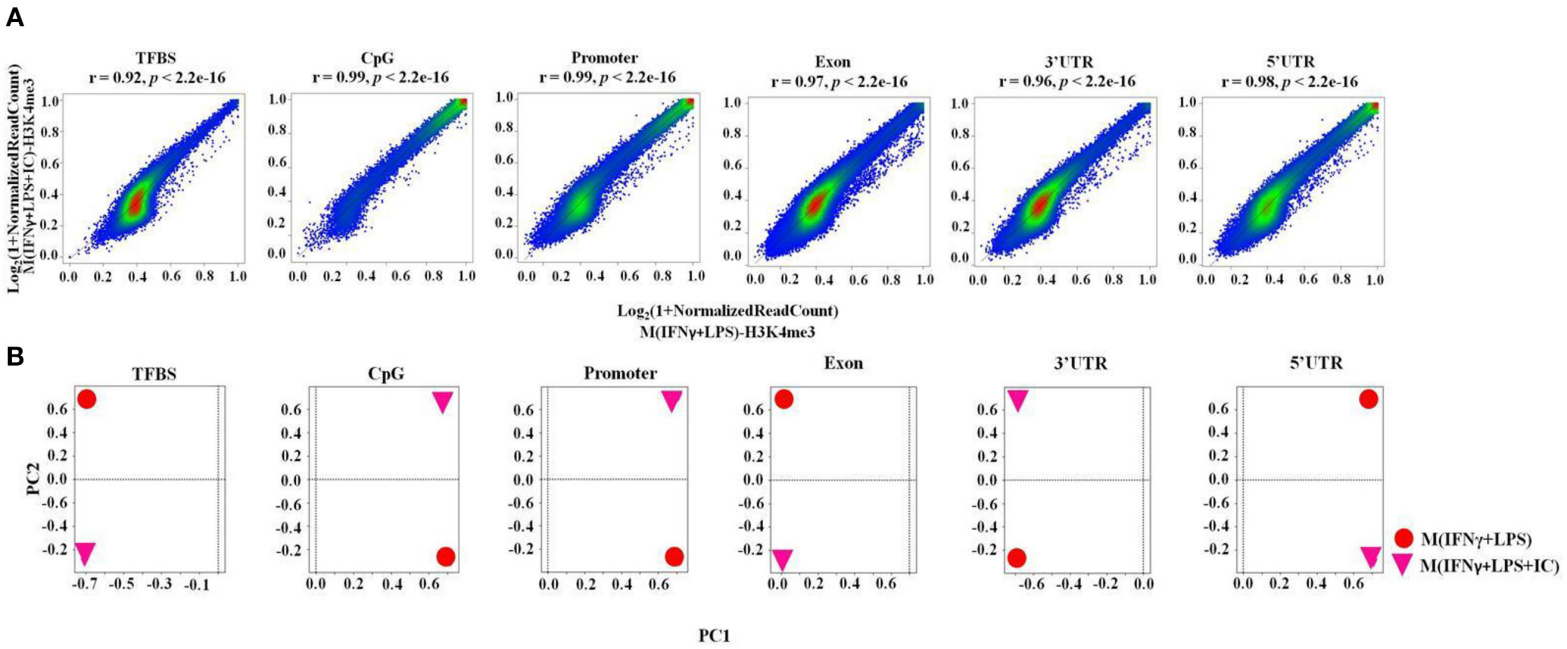
The epigenomic correlation between M(IFNγ+LPS) and M(IFNγ+LPS+IC). **(A)** A scatter plot from EpiMINE showing the epigenomic correlation between M(IFNγ+LPS) and M(IFNγ+LPS+IC) in the *cis*-regulatory elements and other regions. **(B)** The PCA plots show the differences in the two top principal components between M(IFNγ+LPS) and M(IFNγ+LPS+IC).

Next, we performed a correlation analysis at the genome-wide level using TCOR in EpiMINE. The datasets with two distinct correlation methods were analyzed using Pearson correlation and principal component analysis (PCA). The Pearson correlation represented by the scatter plot showed that the M(IFNγ+LPS) and M(IFNγ+LPS+IC) were highly correlated with a Pearson score of more than 0.90, where the *cis*-regulatory elements include transcription factor binding sites (TFBS), CpG islands and promoter regions, and the non-*cis*-regulatory element regions are exons, 3'UTRs, 5'UTRs (Figure 4A) and introns (data not shown). However, the PCA plot revealed that the two top principal components between M(IFNγ+LPS) and M(IFNγ+LPS+IC) in all *cis*-regulatory regions, exons, 3'UTRs and 5'UTRs, are distinct (Figure 4B). These results demonstrated that M(IFNγ+LPS) and M(IFNγ+LPS+IC) are epigenetically highly correlated. More importantly, they show distinctive profiles in H3K4me3 enrichment that may result in differences in gene expression between these two phenotypically distinct effector macrophages.

### Higher H3K4me3 Enrichment in the *cis*-Regulatory Elements in M(IFNγ+LPS+IC) Than M(IFNγ+LPS)

**Figure 5:**
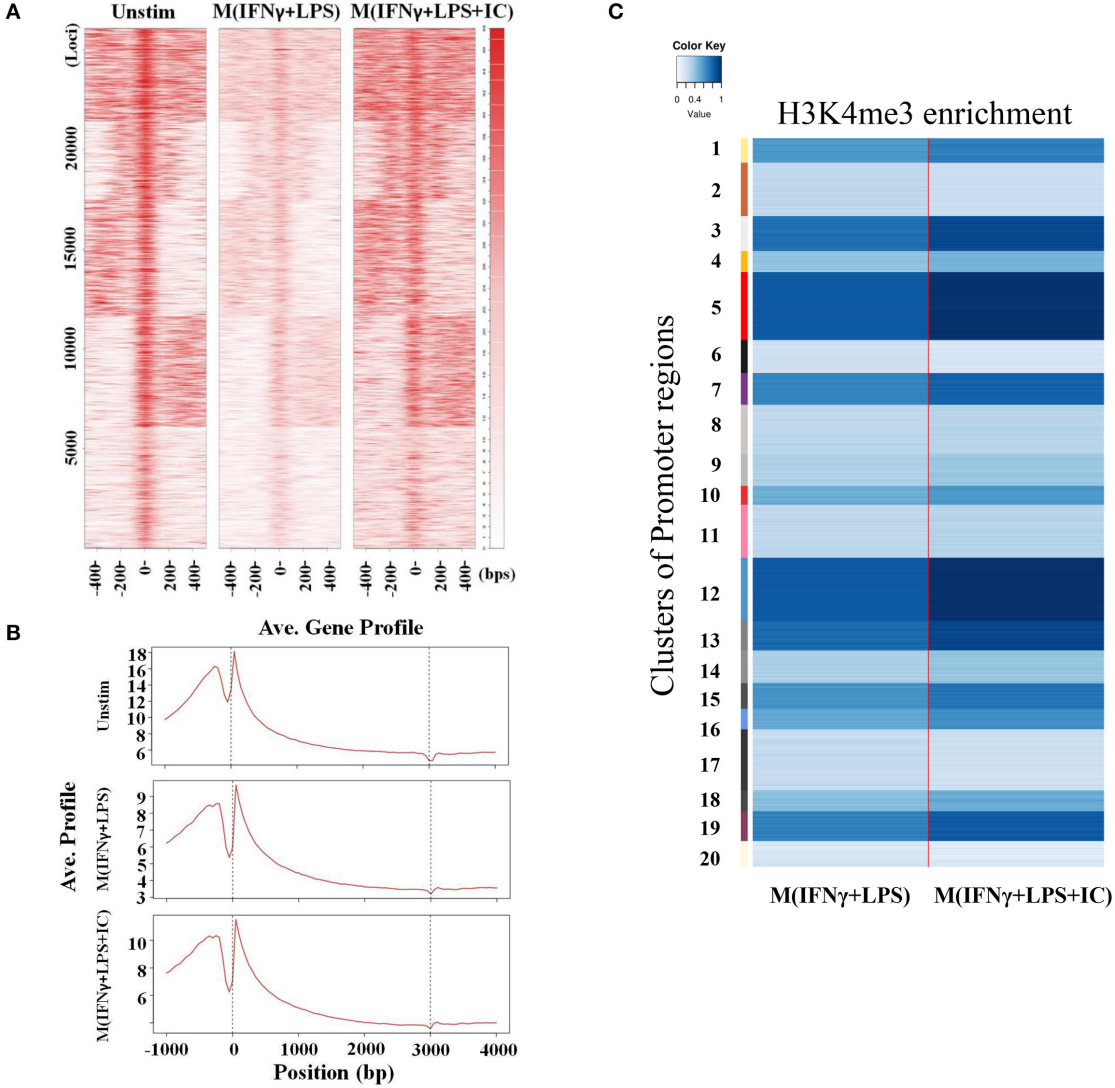
Enrichment of H3K4me3 in *cis*-regulatory regions. **(A)** ChIP-seq density heatmap of H3K4me3 enrichment at the TSSs and the 1 kb region nearby. **(B)** ChIP-seq profiling of H3K4me3 enrichment over a 5 kb window around TSSs. **(C)** Quantification of H3K4me3 enrichment within the promoter regions between M(IFNγ+LPS) and M(IFNγ+LPS+IC).

We focused on specific genomic regions of the *cis*-regulatory elements, including the promoters and TFBS. A heatmap showed H3K4me3 enrichment within the TSSs and 1 kb near the TSSs (Figure 5A). M(IFNγ+LPS+IC) had increased global H3K4me3 enrichment signals when compared with those of M(IFNγ+LPS) (Figure 5A). H3K4me3 peaks at the promoter regions and the TFBS were observed (data not shown). The ChIP-seq peaks were analyzed by CEAS to obtain the average gene profiles of H3K4me3 within the TSSs and the nearby 5 kb. The average gene profiles of all three types of macrophages showed a similar pattern of H3K4me3 enrichment (Figure 5B). From previous results, M(IFNγ+LPS) showed higher percentages of H3K4m3 enrichment distribution in the promoter regions of <1,000 bp than M(IFNγ+LPS+IC) (Figure 3C). However, when QIRI was used to cluster H3K4me3 enrichment regions by k-mean, the results revealed twenty clusters (the presence to absence range was represented with values ranging from 0 to 1), and at the promoter regions, the enrichment of H3K4me3 was higher in M(IFNγ+LPS+IC) than in M(IFNγ+LPS) in all clusters (Figure 5C). These results suggest that H3K4me3 enrichment profiles of M(IFNγ+LPS+IC) in the promoter regions correlated with global H3K4me3 enrichment, and implies that M(IFNγ+LPS+IC) with increased enrichment of H3K4me3 in the promoter regions may have more active gene transcription than M(IFNγ+LPS).

### H3K4me3 Enrichment in Regulatory Regions of Genes Uniquely Upregulated or Downregulated in M(IFNγ+LPS+IC)

**Figure 6:**
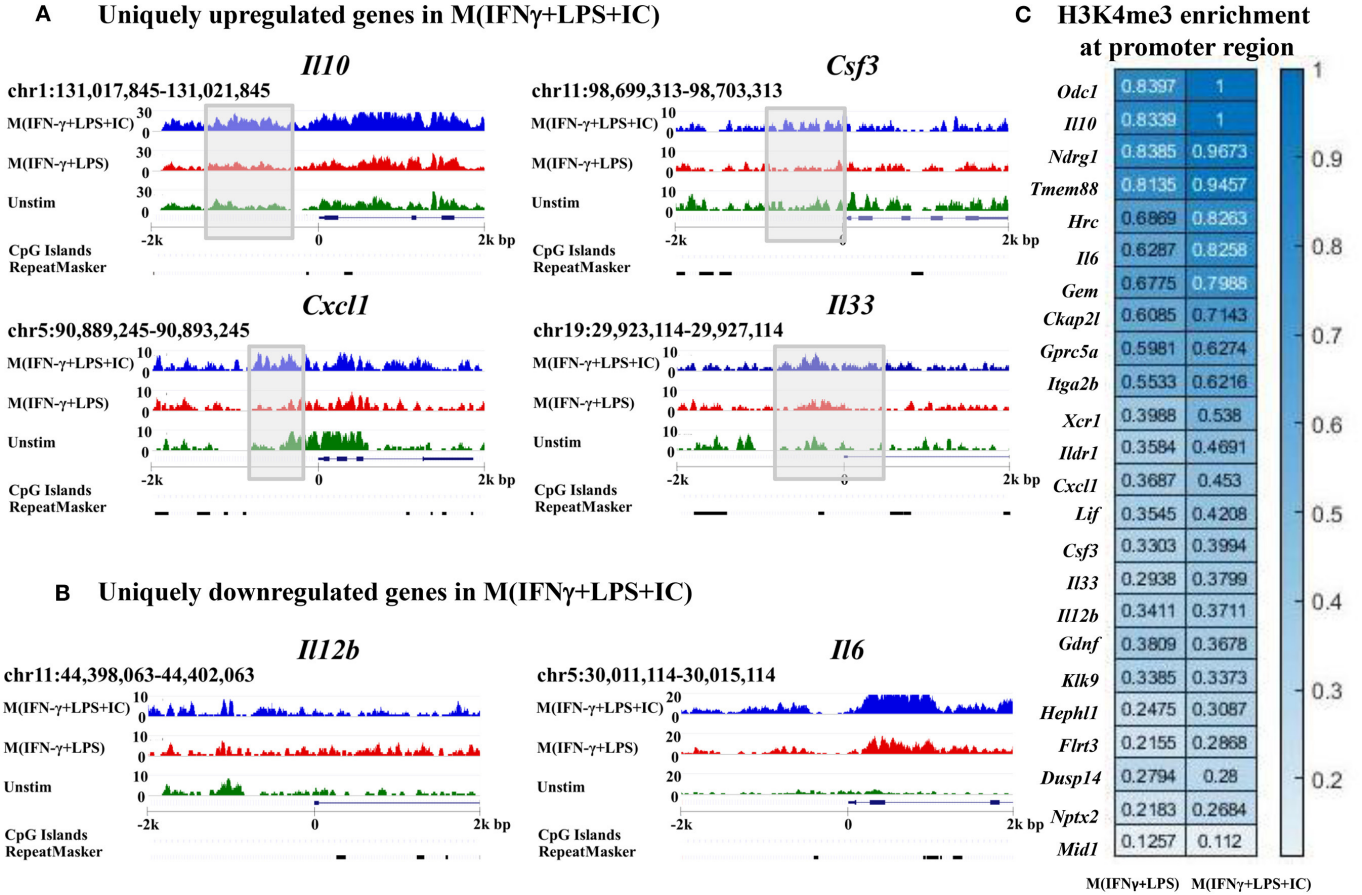
Enrichment of H3K4me3 in the target loci of M(IFNγ+LPS+IC). IGV was used to compare M(IFNγ+LPS+IC)-related loci of uniquely upregulated genes **(A)** and uniquely downregulated genes **(B)** with H3K4me3 enrichment. **(C)** Quantification of H3K4me3 enrichment in the promoter regions of the target genes between M(IFNγ+LPS) and M(IFNγ+LPS+IC).

To link the H3K4me3 profiles with the transcription of the genes, IGV was used to visualize the targeted loci. We investigated H3K4me3 enrichment of M(IFNγ+LPS+IC) in uniquely upregulated genes, including *Il10, Cxcl1, Csf3*, and *Il33*, and uniquely downregulated genes, including *Il12b* and *Il6*, as reported by RNA-seq (11). The results showed increased H3K4me3 enrichment in the uniquely upregulated genes *Il10, Cxcl1, Csf3*, and *Il33* in M(IFNγ+LPS+IC) when compared with that of M(IFNγ+LPS) (Figure 6A). In contrast, the H3K4me3 enrichment in uniquely downregulated genes in M(IFNγ+LPS+IC), *Il12b* and *Il6*, was not different between M(IFNγ+LPS+IC) and M(IFNγ+LPS) (Figure 6B). The quantification of H3K4me3 enrichment by QIRI showed that most of the genes induced during LPS and IC stimulation described by Fleming et al. from RNA-seq data (11) had increased enrichment when compared to those of M(IFNγ+LPS) (Figure 6C). Overall, the H3K4me3 enrichment correlated well with the uniquely upregulated genes in M(IFNγ+LPS+IC), suggesting that the active histone mark H3K4me3 plays active roles in regulating the expression of genes in unique to M(IFNγ+LPS+IC).

### KEGG Pathway Analysis of the Differentially Enriched H3K4me3 Genes in M(IFNγ+LPS+IC) Revealed Key Pathways in the Immune Responses

**Figure 7:**
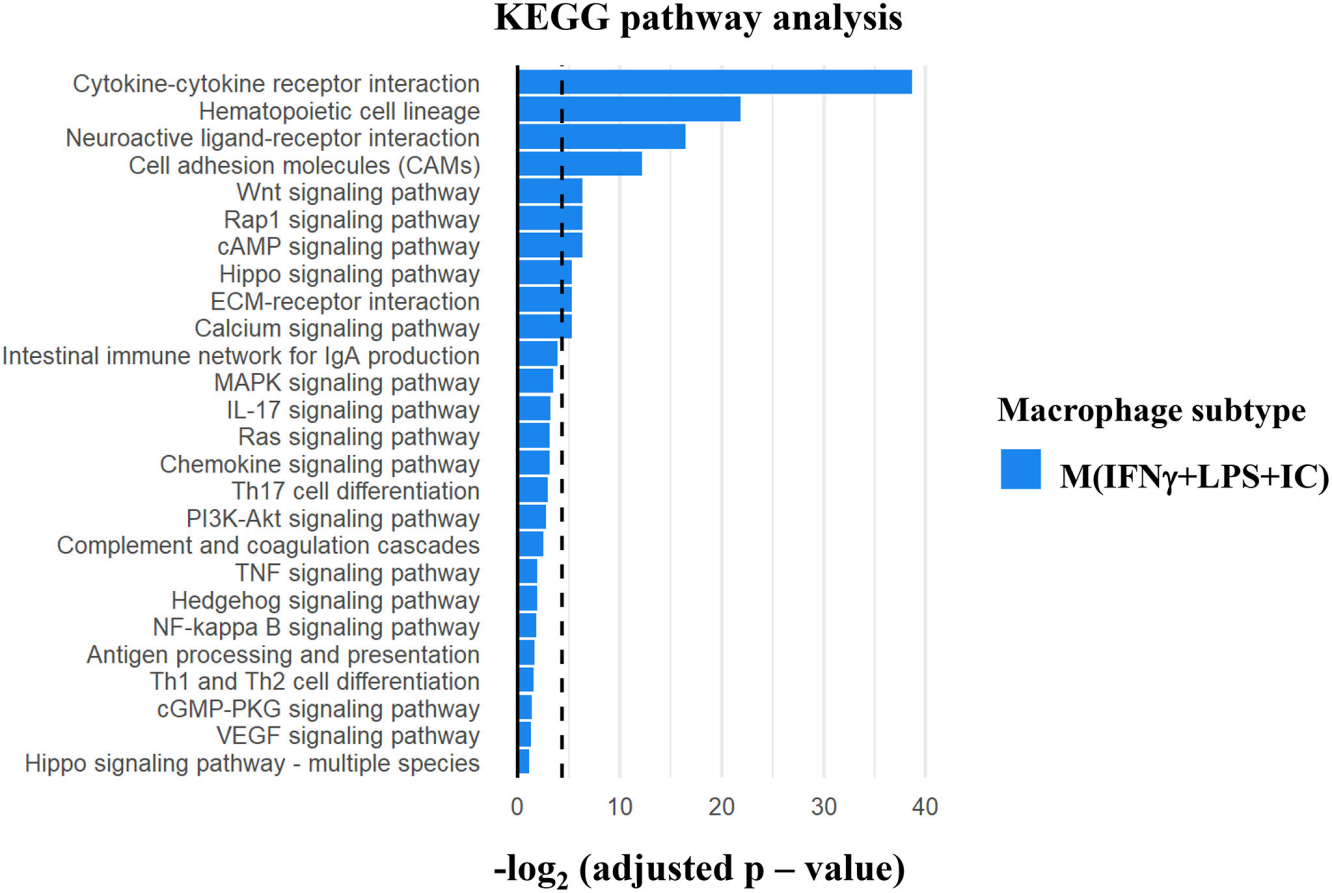
KEGG pathway analysis of the differentially H3K4me3-enriched genes in M(IFNγ+LPS+IC). Gene Ontology analysis using KEGG pathway analysis displayed the significant pathways with differential H3K4me3 enrichment genes in M(IFNγ+LPS+IC). The bar plot showed the ranking of pathways correlated to Immune system, Signal transduction and Signaling molecule (dashed line represents adjusted *p*-value cut-off at 0.05) in M(IFNγ+LPS+IC).

Gene ontology analysis using KEGG pathways was performed to investigate the possible regulatory molecules/signaling pathways revealed by ChIP-seq data in M(IFNγ+LPS+IC). We used genes that showed unique H3K4me3 enrichment in M(IFNγ+LPS+IC) and filtered KEGG pathways for the immune system, signal transduction and signaling molecules to obtain the interaction profiling data. We found 10 significantly enriched pathways in M(IFNγ+LPS+IC) (Figure 7). Among these pathways, the cytokine-cytokine receptor interaction pathway was highly enriched. Cytokines are potent signaling molecules that can activate macrophages to change the phenotype and are crucial for intercellular regulation (29). From the enrichment quantification data, M(IFNγ+LPS+IC) exhibited increased H3K4me3 enrichment in most cytokine genes (Figures 5C, 6C) in the cytokine-cytokine receptor interaction pathway. M(IFNγ+LPS+IC) also showed significantly enriched cell adhesion molecules, RAP1 and cAMP signaling pathways. This result suggests that increased H3K4me3 enrichment may regulate gene expression by inducing downstream signaling pathways that are crucial for functions, polarization and plasticity of M(IFNγ+LPS+IC).

### Adoptive Transfer of M(IFNγ+LPS+IC) Affected Cytokine Profiles in a Mouse LPS-Induced Endotoxemia Model

**Figure 8:**
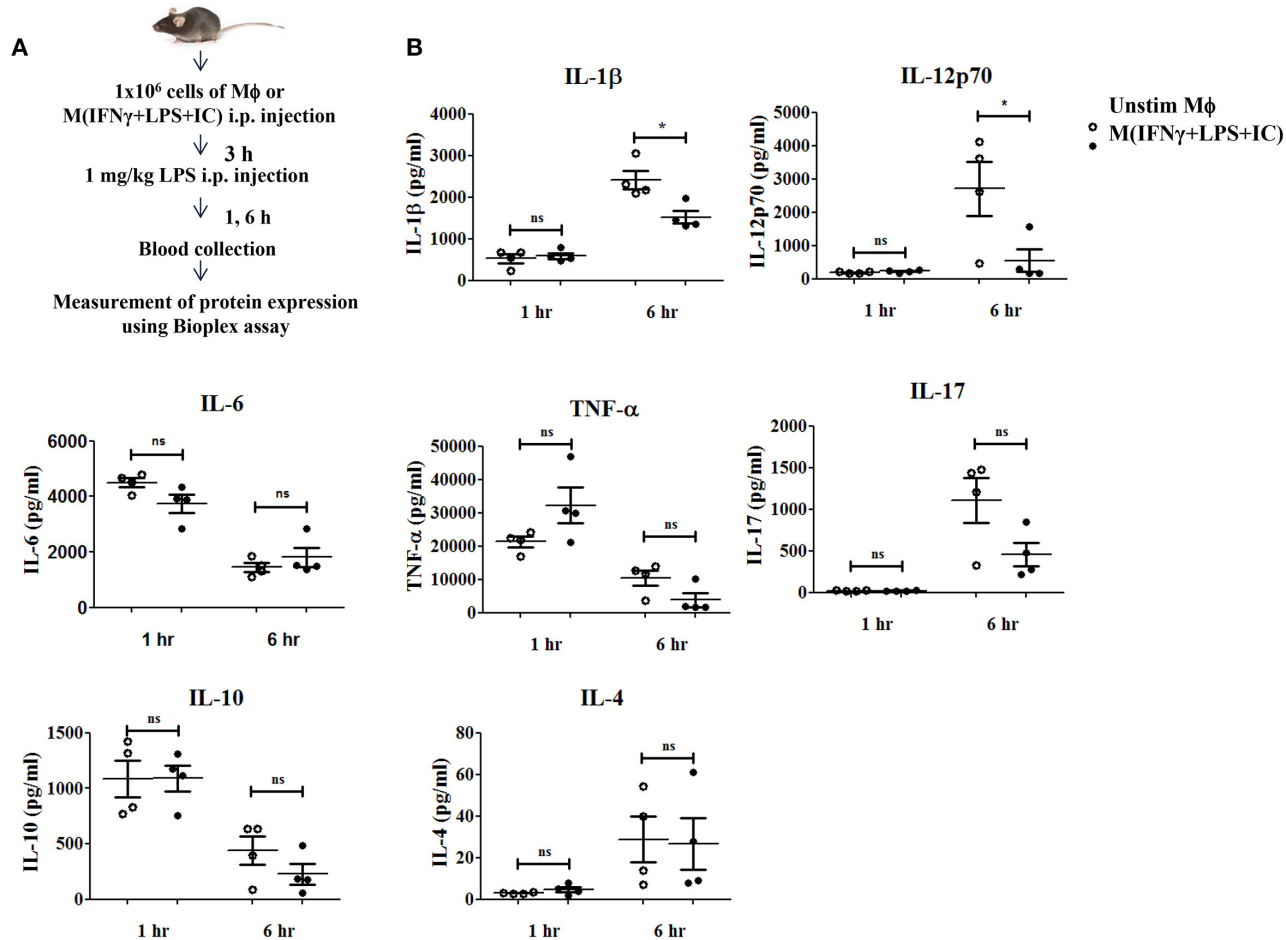
The therapeutic application of M(IFNγ+LPS+IC) in a mouse endotoxemia model. **(A)** The protocol used for the adoptive transfer of M(IFNγ+LPS+IC) in the endotoxemia model. **(B)** Blood sera at 1 and 6 h after LPS challenge from mice with adoptive transfer of unstimulated macrophages or M(IFNγ+LPS+IC) were subjected to Bioplex cytokine assays for IL-6, IL-12p70, IL-1β, TNF-α, IL-17, IL-10, and IL-4. ^*^Statistical significance at *p* < 0.05. The results represent the mean ± SEM of each group (*n* = 4).

A previous study reported that mice receiving an adoptive transfer of M(IFNγ+LPS+IC) produced a high level of IL-10, resulting in decreased disease severity in autoimmune diseases such as experimental autoimmune encephalomyelitis (EAE) (8) and reduced mortality in sepsis (11). To evaluate the impact of M(IFNγ+LPS+IC) *in vivo*, we tested the effect of adoptive transfer of M(IFNγ+LPS+IC) on the systemic cytokine profiles in an LPS-induced endotoxemia model (Figure 8A). The production of the pro-inflammatory cytokines IL-1β and IL-12p70 was significantly decreased at 6 h after LPS challenge in mice receiving adoptive transfer of M(IFNγ+LPS+IC) compared to those receiving unstimulated macrophages (Figure 8B). In contrast, the levels of other inflammatory cytokines, IL-6, TNF-α and IL-17, were not significantly different between the two groups (Figure 8B). Furthermore, for the anti-inflammatory cytokine IL-10 and the Th2 cytokine IL-4, there was no difference between the two groups (Figure 8B). The cytokine profiles revealed that the adoptive transfer of M(IFNγ+LPS+IC) in mice with LPS-induced endotoxemia before LPS administration has a systemic impact on some pro-inflammatory cytokines but no detectable influence on IL-10.

## Discussion

The population diversity and functional plasticity of macrophages are important characteristics of macrophages that play an important role in several diseases (3, 30). LPS in the presence of immune complex stimulates macrophages to become regulatory effector macrophages that produce copious amounts of the anti-inflammatory cytokine IL-10 and reduced amounts of IL-12 (31). Extensive gene expression profiles of M(LPS+IC) have been reported, showing that they are a distinctive subset of activated macrophages (11). It is well-known that macrophages have functional plasticity and can switch phenotypes between M1 or M(LPS) and M2 or M(IL-4). However, no study has reported whether M(LPS) can be reactivated to M(LPS+IC). This is an important question in light of the therapeutic implications of M(LPS+IC) in septicemia and autoimmune diseases such as EAE (8, 11).

To induce macrophages that produce high level of IL-10, IC was used in this study as a “reprograming” stimulus but other stimuli such as prostaglandin E2 and adenosine were reported to yield similar phenotypes when applied to M(LPS) (11). Previously, we characterized in details whether IC alone can induce cytokine production in macrophages or M(IFNγ) and reported that IC alone was not sufficient to induce IL-10, IL-6, TNFα production in IFNγ-primed macrophages (13). This finding highlighted the importance of TLR-mediated stimulation in M(IFNγ+LPS+IC).

IFNγ is a key cytokine in activating macrophages. It has long been reported that stimulating macrophages with LPS together with IFNγ resulted in synergistic effect in inducing anti-microbicidal effector function of macrophages (32, 33). However, detailed transcriptomic analysis revealed that IFNγ priming of murine macrophages repressed subsets of pro-inflammatory genes and prevented the recruitment of neutrophils (34). When the optimal condition to induce IL-10 producing macrophages was examined, it was found that priming yielded better regulatory phenotypes, i.e., increased IL-10 production and decreased IL-12p70 production, of macrophages (8). In this study, it was found that IFNγ prevented the reverse polarization of M(IFNγ+LPS) to M(LPS+IC). Without the priming, it was possible to re-polarize M(LPS) to become M(LPS+IC). This result led us to explore the involvement of epigenetic regulation in M(IFNγ+LPS) and M(IFNγ+LPS+IC).

Furthermore, we uncovered that at least *in vitro* re-stimulation of M(LPS) with LPS in the presence of IC requires a washout period of a minimum of 48 h between the two opposing stimuli. If the washout period was shorter (2–24 h), the ex-M(LPS) could not produce IL-10 at high level with reducing IL-12 upon re-stimulation with LPS and IC, as observed in M(LPS+IC). In contrast, the re-stimulated M(LPS) had decreased levels of most cytokines associated with M(LPS+IC). Some of the signaling pathways downstream of TLR4 and FcR (MAPK p38, SAPK/JNK, NF-κB) were all reduced, which correlated with this unresponsive phenotype. IL-10 production in macrophages stimulated with TLR/LPS is regulated by the MAPK and NF-κB signaling pathways (10). LPS tolerance of macrophages describes the phenomenon where macrophages become unresponsive to successive rounds of LPS stimulation (35). The shorter resting time that resulted in the failure of re-polarization may be partially explained by LPS tolerance. After 2 days of resting, re-stimulation of M(LPS) with LPS/IC rescued all early signal transduction and the production of high IL-10 and low IL-12.

A previous study demonstrated that ERK activation and histone modification by phosphorylation of serine 10 on H3 plays a key role in regulating IL-10 expression in M(LPS+IC) (20). In this study, we focused on H3K4me3 as a representative of active histone marks because it is the most dynamic epigenetic modification in activated macrophages found at gene promoters (36). H3K4me3 are histone marks associated with actively transcribed genes (37). By ChIP-seq analysis, we found that compared to M(IFNγ+LPS), M(IFNγ+LPS+IC) had high H3K4me3 enrichment globally, especially in the *cis*-regulatory elements (Figures 3A,B, 5A). The quantification of enrichment in the promoter regions showed high H3K4me3 enrichment in M(IFNγ+LPS+IC) in all clusters and most of the genes that were found to be induced in M(LPS+IC) from RNA-seq (11).

Interestingly, the results also showed an increase in H3K4me3 enrichment in the promoter regions of most of the target loci that are uniquely upregulated in M(IFNγ+LPS+IC), i.e., *Il10, Cxcl1, Csf3*, and *Il33*. Our study revealed for the first time that H3K4me3 in M(IFNγ+LPS+IC) is another epigenetic modification that macrophages use to regulate IL-10 expression. In addition, the promoters of *Odc1* and *Ndgr1* showed a marked difference between M(IFNγ+LPS) and M(IFNγ+LPS+IC). *Odc1* encodes ornithine decarboxylase, a rate-limiting enzyme of the polyamine biosynthesis pathway. Ornithine decarboxylase is reported in macrophages to regulate M1, and specific deletion of this gene in macrophages results in hyperactivation of M1 and exacerbates colitis (38). Furthermore, ornithine decarboxylase modifies histones that impinge upon M1 gene expression (39). Our results imply that M(IC) may regulate *Odc1* expression by increasing H3K4me3 in the promoter region. *Ndgr1* encodes N-myc downstream regulated 1, which is a member of the NDGR family. Interestingly, *Ndgr1* KO mice exhibit impaired M1/M2-type macrophage differentiation, and the expression of NDGR1 was found in tumor-infiltrating macrophages in renal cancer (40, 41).

For the downregulated genes in M(IFNγ+LPS+IC), *Il6* and *Il12b* showed no significant difference in H3K4me3 enrichment between M(IFNγ+LPS) and M(IFNγ+LPS+IC). This result suggests that other histone modifications that override H3K4me3 enrichment at promoter regions, may play dominant roles in transcriptional regulation.

For the epigenomic correlation, TCOR in EPIMINE were used to analyze the correlation between M(IFNγ+LPS) and M(IFNγ+LPS+IC). To our surprise, these two phenotypes of macrophages were highly correlated in all *cis*-regulatory elements and in other regions, even though the PCA plots showed a difference in the two top principal components between M(IFNγ+LPS) and M(IFNγ+LPS+IC).

We further investigated the possible regulatory molecules/signaling pathways using KEGG pathway analysis. H3K4me3 enrichment was found in regulatory regions of genes associated with cytokine-cytokine interactions, hematopoietic cell lineage, cell adhesion molecules, the Wnt signaling pathway and the RAP1 signaling pathway. All of these pathways have been demonstrated to be involved in macrophage function and activation. RAP1 signaling regulates downstream signaling of MAPK and PI3K/Akt (42) that is crucial for M(LPS+IC) polarization and plasticity. The Wnt/β-catenin pathway is linked to alternatively activated macrophages that contribute to kidney fibrosis (43, 44). Therefore, trimethylation of H3K4 may be a key epigenetic mechanism that regulates gene expression during macrophage activation.

Epigenetics includes a variety of regulatory mechanisms, including many types of histone modifications, both permissive and repressive, DNA methylation, and noncoding RNA-mediated regulation. In fact, many types of epigenetic modification have been reported during macrophage activation and function (45, 46). Therefore, we cannot rule out that other epigenetic regulations may also play important roles in M(IC) and their plasticity. How H3K4me3 is regulated in M(IFNγ+LPS) and M(IFNγ+LPS+IC) is currently not well-understood. Identification of H3K4 methyltransferase or demethylase in these subsets of macrophages will help us better understand this process and needs further investigation.

Finally, in the translational approach, we investigated the cytokine profiles in the blood serum of mice after adoptive transfer of M(IFNγ+LPS+IC) in a mouse sepsis model. Previously, it was reported that M(LPS+IC) reduce the lethality of LPS-induced endotoxemia, but the impact on the systemic cytokine profiles has not been explored (11). We found that mice with adoptively transferred M(IFNγ+LPS+IC) had specifically decreased IL-1β and IL-12p70 levels in blood serum when compared to mice with adoptively transferred unstimulated macrophages (Figure 8B). Surprisingly, the IL-10 level was not significantly different between the two groups (Figure 8B). In the EAE model, adoptive transfer of M(IFNγ+LPS+IC) reduced the severity of Th1/Th17-mediated EAE, and the impact was seen in T cells that produce IL-10 and IL-4 upon re-stimulation (8). Currently, it is not known how adoptive transfer of M(IFNγ+LPS+IC) decreases IL-1β and IL-12p70 levels in blood serum. It is possible that via IL-10, M(IFNγ+LPS+IC) directly dampen pro-inflammatory cytokine production. Alternatively, M(IFNγ+LPS+IC) may indirectly influence IL-1β and IL-12p70 by other mechanisms.

In conclusion, our study demonstrated that M(LPS) and M(LPS+IC) have functional plasticity at least *in vitro*. The priming with IFNγ and the resting time between the two opposing stimuli are the keys for the recovery of the phosphorylation of important downstream signaling pathways. We also showed that active histone H3K4me3 marks were increased to a greater extent in M(IFNγ+LPS+IC) than M(IFNγ+LPS). The significantly enriched pathways in M(IFNγ+LPS+IC) are associated with cytokines and gene expression in M(IFNγ+LPS+IC). Moreover, we investigated the therapeutic application of M(IFNγ+LPS+IC) that dampen the production of pro-inflammatory cytokines in a mouse sepsis model. Hence, the manipulation of the epigenetic regulation by H3K4me3 may help modulate M(IFNγ+LPS+IC) polarization and plasticity for future therapeutic applications.

## Data Availability Statement

The datasets generated for this study can be found in the NCBI Gene Expression Omnibus (GEO) and are accessible through GEO accession number GSE129284 (https://www.ncbi.nlm.nih.gov/geo/query/acc.cgi?acc=GSE129284).

## Ethics Statement

All procedures were reviewed and approved by the Chulalongkorn University animal care and use protocol committee (CU-ACUP 024/2558).

## Author Contributions

VR designed and performed all experiments, analyzed all data and prepared all figures and the manuscripts. PB helped in performing ChIP-seq experiment. Y-WL, MP, and PK analyzed ChIP-seq data and the KEGG pathways. AL designed experiments and analyzed the sepsis data. TP designed all experiments, analyzed all data and prepared the manuscript.

### Conflict of Interest

The authors declare that the research was conducted in the absence of any commercial or financial relationships that could be construed as a potential conflict of interest.
