# Supplementary material for: Profile of Histone H3 Lysine 4 Trimethylation and the Effect of Lipopolysaccharide/Immune Complex-Activated Macrophages on Endotoxemia
Source: Front Immunol. 2020 Jan 10;10:2956. doi: 10.3389/fimmu.2019.02956 (PMC6965496; doi:10.3389/fimmu.2019.02956)
Supplement: Supplementary file 1 [file Data_Sheet_1.docx]

**Supplementary Information**

Profile of Histone H3 Lysine 4 Trimethylation and the Effect of Lipopolysaccharide/Immune Complex-Activated Macrophages on Endotoxemia

Ruenjaiman et al.


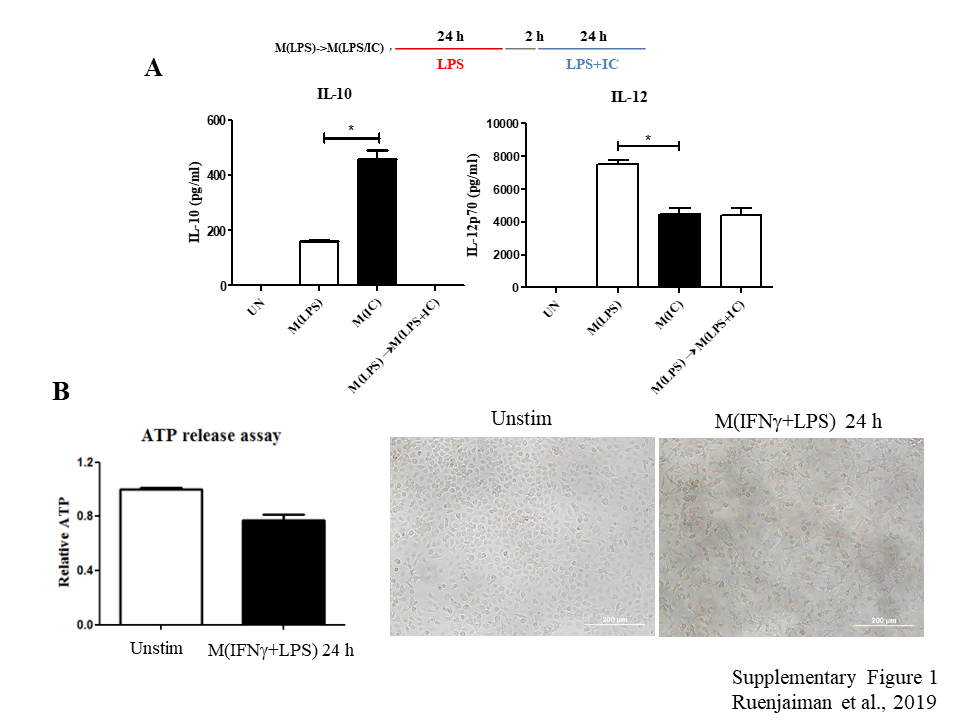


Supplementary Figure 1

(A) The protocol used for stimulation of M(LPS) and M(LPS+IC) is shown with the washout period of 2 h. IL-10 and IL-12p70 in the culture supernatant harvested from cells treated as described were analyzed by ELISA. The results indicate the means ± SD of triplicates determined from 3 independent experiments. * indicates a significant difference at p<0.05.

(B) BMDMs were stimulated with LPS for 24 h and the cell viability assay was performed. Images of cells observed under inverted microscope.


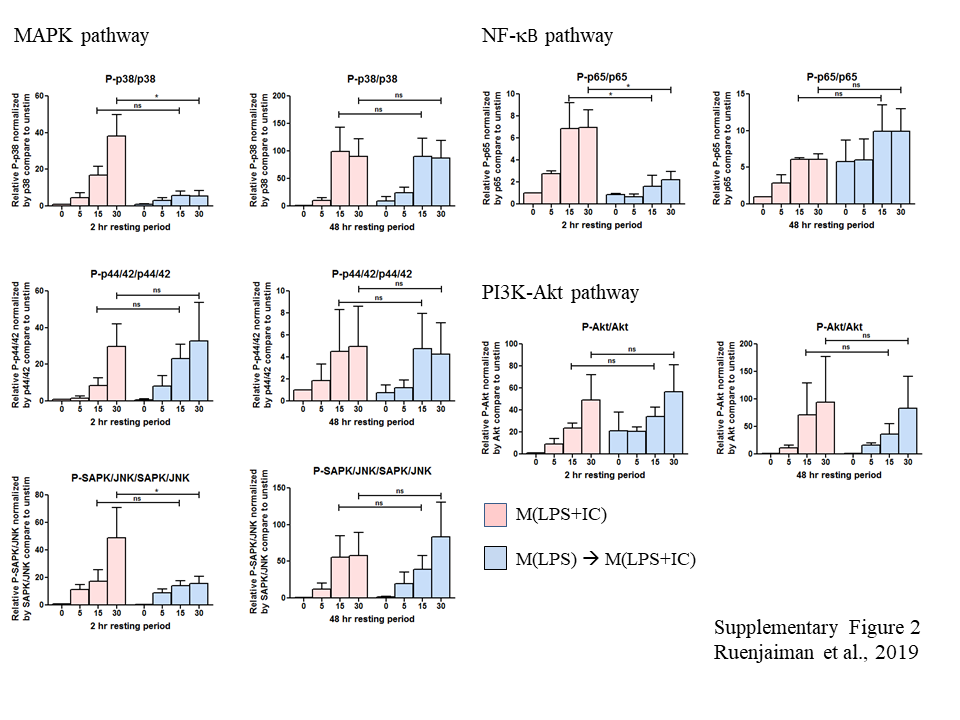


Supplementary Figure 2

Band densities of Western blots shown in Figure 2 were subjected for analysis by ImageJ software. The normalized band densities are shown with the washout period of 2 h and 48 h between M(LPS) and M(LPS+IC). ns; not significance, * indicated statistical significant with *p*<0.05.
